# Identifying Biomarkers to Predict the Progression and Prognosis of Breast Cancer by Weighted Gene Co-expression Network Analysis

**DOI:** 10.3389/fgene.2020.597888

**Published:** 2020-12-17

**Authors:** Gengsheng Shi, Zhenru Shen, Yi Liu, Wenqin Yin

**Affiliations:** ^1^Department of Clinical and Public Health, School of Health and Rehabilitation, Jiangsu College of Nursing, Jiangsu, China; ^2^Department of Cardiothoracic Surgery, The Second People’s Hospital of Huai’an, Huai’an, China

**Keywords:** breast cancer, WGCNA, progression, cell cycle, prognosis

## Abstract

Breast cancer (BC) is the leading cause of cancer death among women worldwide. The molecular mechanisms of its pathogenesis are still to be investigated. In our study, differentially expressed genes (DEGs) were screened between BC and normal tissues. Based on the DEGs, a weighted gene co-expression network analysis (WGCNA) was performed in 683 BC samples, and eight co-expressed gene modules were identified. In addition, by relating the eight co-expressed modules to clinical information, we found the blue module and pathological stage had a significant correlation (*r* = 0.24, *p* = 1e–10). Validated by multiple independent datasets, using one-way ANOVA, survival analysis and expression level revalidation, we finally screened 12 hub genes that can predict BC progression and prognosis. Functional annotation analysis indicated that the hub genes were enriched in cell division and cell cycle regulation. Importantly, higher expression of the 12 hub genes indicated poor overall survival, recurrence-free survival, and disease-free survival in BC patients. In addition, the expression of the 12 hub genes showed a significantly positive correlation with the expression of cell proliferation marker Ki-67 in BC. In summary, our study has identified 12 hub genes associated with the progression and prognosis of BC; these hub genes might lead to poor outcomes by regulating the cell division and cell cycle. These hub genes may serve as a biomarker and help to distinguish different pathological stages for BC patients.

## Introduction

In 2018, there are approximately 2.1 million new cases of breast cancer (BC) and 630,000 deaths worldwide (Bray et al., 2018). Although adjuvant therapies have reduced BC-related mortality, up to 25% of patients will develop tumor relapse (Early Breast Cancer Trialists’ Collaborative Group et al., 2012; Howlader et al., 2014). The mortality of BC is largely due to recurrent tumors (Berry et al., 2005). BC patients with higher clinical stage are more likely to recurrence and have worse prognosis (Garcia-Murillas et al., 2015). Genetic mutations have a key role in the progression of BC. About 20% of triple-negative BC patients have BRCA mutation, while BRCA mutations are rarely found in the healthy population (Trainer et al., 2011). Over 30% of BC patients have overexpressed HER2. Ki-67 was reported to be associated with disease-free survival of BC (Kontzoglou et al., 2013). BRAF mutations were present in over 3% of metastatic BC patients (Cejalvo et al., 2016). Although there have been great advances in the treatment of BC, the ability to treat advanced BC is still limited due to the lack of precise molecular targets (Meng et al., 2016). Therefore, more novel candidate genes are needed to improve early diagnosis and treatment decisions.

Many studies have suggested that genes were involved in tumor progression and prognosis (van Kessel et al., 2018; McFaline-Figueroa et al., 2019). Gene expression profiles such as microarray and RNA-sequencing are common ways to determine biomarkers related to progression of various cancers (Dahinden et al., 2010; Gerlinger et al., 2014). However, most published studies have focused on the screening of differentially expressed genes (DEGs), ignoring the high connection between genes, although genes with similar expression patterns may be functionally related (Tavazoie et al., 1999). Therefore, it is very limited to merely focus on DEGs between normal and tumor cells, and more attention should be paid to combination of gene expression pattern and clinical features, such as tumor stage, histological grade, invasiveness, etc.

Co-expression networks are widely used to decipher disease mechanisms and provide a systematic view of dysregulation pathways (Nayak et al., 2009). The basic theory of co-expression analysis is that co-expressed genes may be functionally related. Weighted gene co-expression network analysis (WGCNA) is an open source tool to perform co-expression analysis based on the theory. WGCNA integrates the expression differences between samples into a higher-order network structure, and clarifies the relationship between genes based on their co-expression profiles. The WGCNA algorithm has been used to investigate the associations between gene modules and clinical indicators in the field of cancer research (Langfelder and Horvath, 2008). Specifically, WGCNA was applied to identify key genes significantly associated with clinical indicators of tumor progression including tumor stage, grade, and metastasis (Chen L. et al., 2017; Chen P. et al., 2017). WGCNA has been used to identify biomarkers related to BC progression in recent publications. Tang et al. screened several prognostic genes including CCNB2, FBXO5, KIF4A, MCM10, and TPX2 using WGCNA (Tang et al., 2018). Another recent study suggested that four hub genes (FAM171A1, NDFIP1, SKP1, and REEP5) were identified as candidate biomarkers for BC (Tian et al., 2020). WGCNA was also used to identify key modules and pathways in BC. Our study intends to use this algorithm to identify biomarkers associated with progression of BC. We try to construct a co-expression network of genes and identify the network hub genes related to the clinical characteristics of BC, and use various databases (GEO, TCGA, and STRING) to verify our results.

## Materials and Methods

### Data Collection

Normalized gene expression data and corresponding clinical information were downloaded from Gene Expression Omnibus (GEO)^1^. Datasets GSE42568 (Cheng et al., 2017) performed on the platform Affymetrix Human Genome U133 Plus 2.0 Array included gene expression profiling of 104 BC and 17 normal breast biopsies. GSE42568 was analyzed to screen differential expressed genes (DEGs). Dataset GSE102484 (Kao et al., 2011) also performed on Affymetrix Human Genome U133 Plus 2.0 Array included 683 BC samples, which was used to perform weighted gene co-expression networks. Dataset GSE20685 (Sabatier et al., 2011b) had gene expression profiles of 327 BC samples. Dataset GSE21653 (Sabatier et al., 2011a) had 266 samples and was used for Ki-67 correlation analysis and module preservation analysis. In addition, 992 BC samples with RNA-seq data were obtained from the Cancer Genome Atlas (TCGA) database. GSE20685 and TCGA were both used for stage validation.

### Screening for DEGs

Normalized gene expression matrix and corresponding annotation files were obtained from GEO database. Firstly, we used the annotation files to annotate the probes. DEGs between normal and tumor breast samples were identified by R package “limma” (Ritchie et al., 2015). The cut-off criteria were the FDR (false discovery rate) < 0.01 and |log2(fold change)| ≥ 1.

### Weighted Gene Co-expression Network Construction

Based on the expression values of all DEGs of 683 BC samples and the corresponding clinical information (GSE102484), the “WGCNA” (Langfelder and Horvath, 2008) R package was used for the co-expression network (Langfelder and Horvath, 2008). Before constructing the co-expression network, outlier samples should be excluded by sample clustering using Pearson’s method. According to the tutorial of WGCNA, we firstly verified the qualification of genes and samples. Then we construct the Pearson correlation matrix, and use the formula amn = |cmn|^β^ (cmn represents the Pearson correlation between genes, amn represents the adjacency between genes, β parameter can amplify the correlation between genes) to obtain the weighted adjacency matrix. The soft threshold power β is determined based on the standard scale-free network. Subsequently, we converted the adjacency relationship into a topological overlap matrix (TOM) (Yip and Horvath, 2007), and hierarchically clustered genes to identify modules containing similar genes. In this study, we selected the minimum size as 30 for the gene dendrogram, selected the cutting line (0.25) for the modular dendrogram, to merge some similar modules.

### Identify Modules With Clinical Significance and Functional Annotations

It is of great biological significance to identify modules most significantly associated with clinical features. Based on the similarity expressed in samples, gene modules with clinical significance were identified by correlation analysis. We selected the gene modules most relevant to clinical features as the modules of interest, and analyzed their correlation with clinical features. In addition, in order to further clarify the potential mechanism of module of interest affecting clinical features, the genes were uploaded to DAVID (Dennis et al., 2003) (The Database for Annotation, Visualization, and Integrated Discovery) for GO function enrichment analysis. False transmission rate (FDR) < 0.01 was considered statistically significant.

### Module Preservation Analysis

Module preservation analysis was conducted to ensure the identified gene modules could also be found in the test network (Langfelder et al., 2011). To evaluate the module preservation, we applied median rank and Zsummary via permutation testing in the “WGCNA” package. Considering the computational complexity involved in the size of our network, the permutation was executed 200 times. According to the threshold set in a previous study (Langfelder et al., 2011), modules with ZSummary scores > 10 indicate preservation, 2–10 indicate weak to moderate preservation, and < 2 indicate no preservation in the permutation. The dataset GSE21653 was used for preservation analysis including 266 BC samples.

### Hub Gene Identification and Validation

Hub genes have a significant correlation with clinical characteristics (Gene significance, GS), and have a high module characterization (Module Membership, MM) in the module. Hub genes, also called key genes, are a group of genes with the highest connectivity, and determine the characteristics of the gene module. There are two ways to identify the key genes in the module according to the official tutorial of WGCNA (Langfelder and Horvath, 2008). The first is to directly determine the key genes based on GS and MM greater than a certain threshold. Specifically, the screening criterion with GS greater than 0.2 and MM of more than 0.8 are often used. The second is to use the “networkScreening” function, which can be used to calculate the weighted *P*-value p.weighted of each gene. Our study chose the first way to identify hub genes. In order to ensure the reliability of the hub genes, we used other independent datasets to validate the expression of hub genes in different tumor stages. We used BC samples from other independent datasets to compare hub gene expression at different pathological stages. We also obtained prognostic data for hub genes and analyzed the survival time of each gene.

## Results

### Screening DEGs in BC Tissue Samples

**FIGURE 1:**
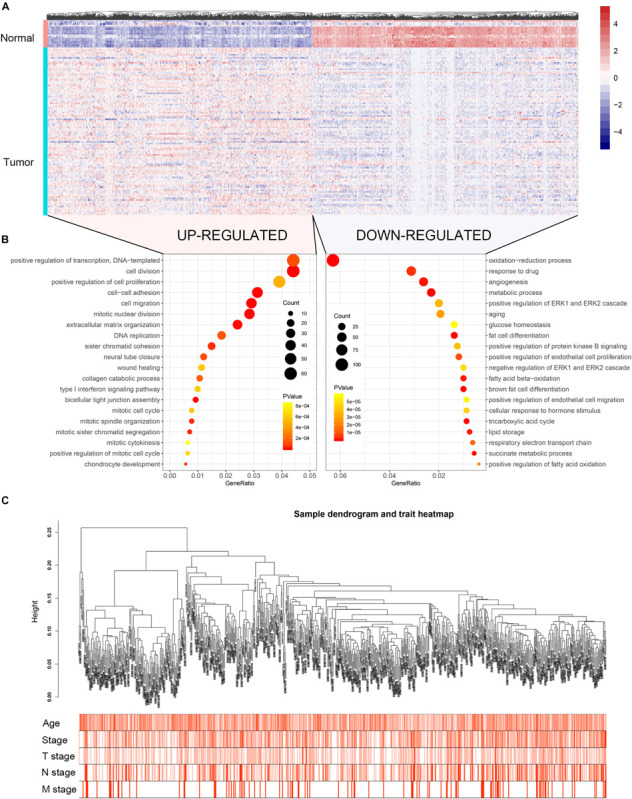
Heatmap of the DEGs, GO functional annotation and clustering dendrogram of 683 tumor samples. **(A)** The heatmap shows the DEGs between 104 BC and 17 normal breast samples based on the dataset GSE42568. **(B)** The bubble plot shows the enriched biological processes of the upregulated genes and downregulated genes. **(C)** The clustering of 683 BC samples based on all DEGs expression and clinical features. The color intensity represents older age and higher stage.

The flow chart of the study is shown in Supplementary Figure S1. When the cut-off criteria is FDR < 0.05 and |log2 (FC)| ≥ 1, 3046 DEGs were screened between 104 BC and 17 normal breast biopsies from dataset GSE42568. The heatmap of all DEGs was shown in Figure 1A. Pathway and functional enrichment analysis showed that the upregulated DEGs were significantly enriched in cell proliferation and migration related pathways, including cell division, positive regulation of cell proliferation, cell–cell adhesion, cell migration etc. The downregulated DEGs were associated with metabolism related pathways, such as metabolic process, glucose homeostasis, and fatty acid beta-oxidation.

### Identifying Co-expression Network and Module Preservation Analysis

**FIGURE 2:**
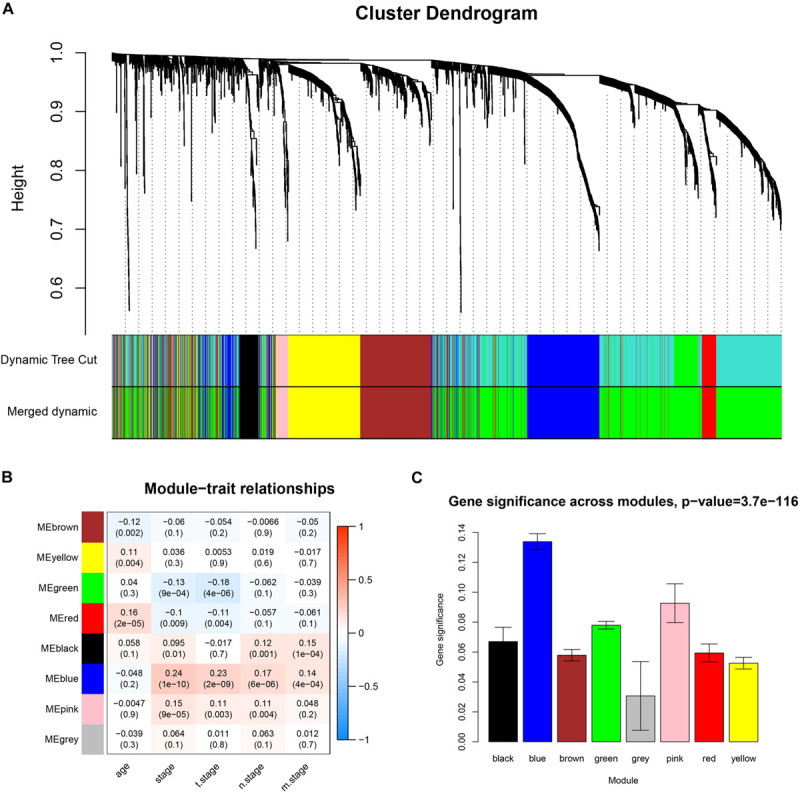
Identifying co-expressed modules and the correlation between modules and the clinical features. **(A)** Dendrogram of all DEGs clustered based on a dissimilarity measure (1-TOM). **(B)** Correlation heatmap showed the correlation coefficients between gene modules and clinical features of BC. **(C)** The bar plot showed the distribution of average gene significance at different modules.

**FIGURE 3:**
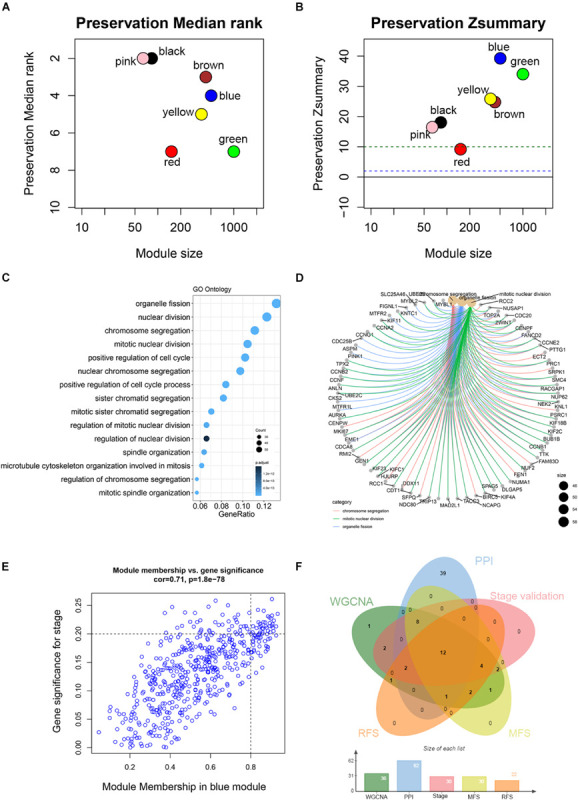
Module preservation analysis and functional enrichment of clinically significant module. **(A)** The medianRank statistics of the module preservation using independent dataset GSE21653. The medianRank of the modules close to zero indicates a high degree of module preservation. **(B)** The Zsummary statistics of the module preservation using independent dataset GSE21653. These horizontal lines represent the Zsummary threshold (*Z* = 2 and *Z* = 10), which is used to indicate strong evidence of preservation (above 10) and low to moderate preservation (above 2). **(C)** Biological processes of genes in the blue module. The *x*-axis is the gene ratio, which is the ratio of enriched genes to the total number of genes in the term. **(D)** The three top-ranked biological processes and the corresponding genes of the blue module. **(E)** Scatter plot shows the gene module membership and gene significance of the blue module. **(F)** Common genes with statistical significance in different methods, including survival, one-way ANOVA, Pearson’s correlation, and co-expression analysis.

Co-expression analysis included 683 BC samples and their complete clinical data and 3046 differential gene expression data. Four outlier samples were excluded after the samples were clustered by correlation analysis (Figure 1C and Supplementary Figure S2A). We used WGCNA R package and classified differential genes with similar expression patterns into different modules by average link clustering. When the soft threshold β was selected as 8, the genes in the network was scale-free (Supplementary Figures S2B–E). Different modules were identified, and the genes in the same module had a similar co-expression trend. A total of 8 modules were identified after the modules with a similar co-expression trend were combined (Figure 2A). The genes in the gray module were not co-expressed (Figures 2A,B). We did module preservation analysis by comparing the identified gene modules above with the test dataset GSE21653 to ensure the repeatability of the modules. As shown in Figures 3A,B, since the Zsummary statistic of the blue module was higher than 10 and the median rank statistic was close to the minimum in the test dataset, the module showed considerable stability.

### Identifying Clinically Significant Gene Modules

The main purpose of WGCNA is calculating the correlation between different modules and clinical features, and identifying the modules most relevant to clinical features, which has important biological significance. We used Pearson’s correlation analysis to calculate the correlation coefficients between different gene modules and clinical features, and found that the blue module and tumor stage (*R* = 0.24, *p* = 1e–10) has the highest correlation, and it also has a significant correlation with the tumor T stage (t.stage, *R* = 0.23, *p* = 2e–9) Figure 1B. The bar plot in Figure 2C also showed that the blue module had the highest gene significance across all modules. Therefore, the blue module is identified as a clinically significant module for further analysis. To investigate the functional role of the 504 genes of the blue module, we performed GO enrichment analysis and found that the biological process mainly focused on cell cycle and cell division (all *p* < 0.01, Figure 3C). Under the threshold of *p* < 0.01, Figure 3C showed the genes included in the three top biological processes including chromosome segregation, mitotic nuclear division, and organelle fission.

### Identification and Validation of Hub Genes

**FIGURE 4:**
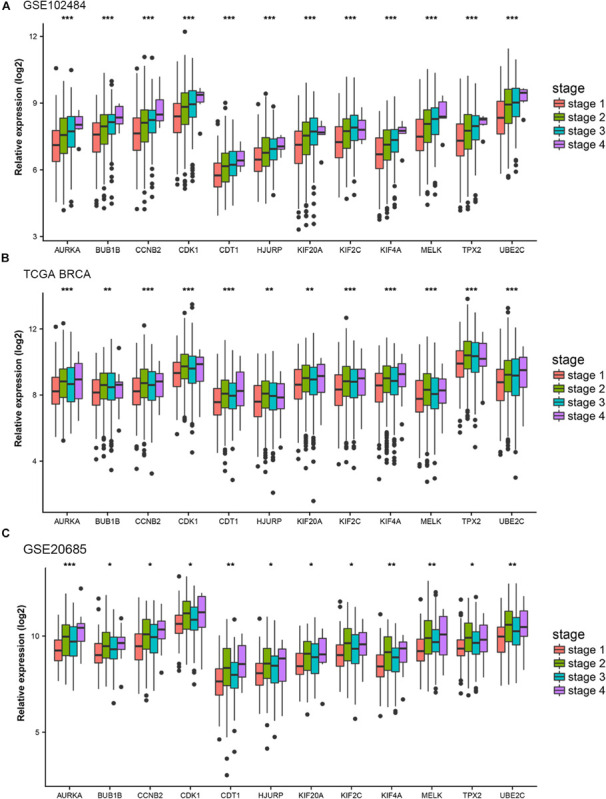
Stage validation of hub gene. **(A)** Relative expression of the 12 hub genes at different stages in the GSE102484. **(B)** Relative expression of the 12 hub genes at different stages in TCGA BRCA. **(C)** Relative expression of the 12 hub genes at different stages in the GSE20685. The medians and dispersions are shown in the boxplot. One-way ANOVA is used to test statistical significance. *p < 0.05, **p < 0.01, ***p < 0.001.

**FIGURE 5:**
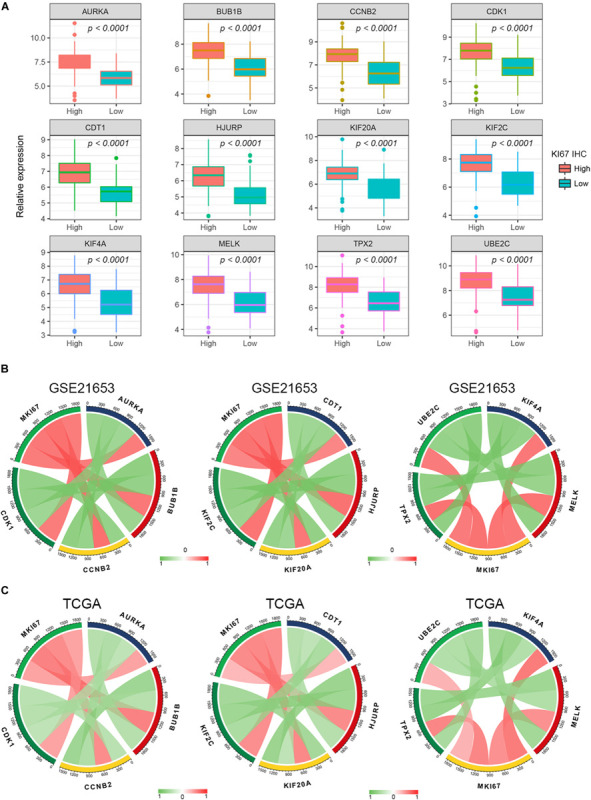
Pearson’s correlation analysis for the expression of Ki67 and the candidate hub genes. **(A)** The gene expression of the hub genes in Ki-67 IHC staining high and low BC samples. **(B,C)** The Pearson’s correlation between the expression of MKi67 and the hub genes.

**FIGURE 6:**
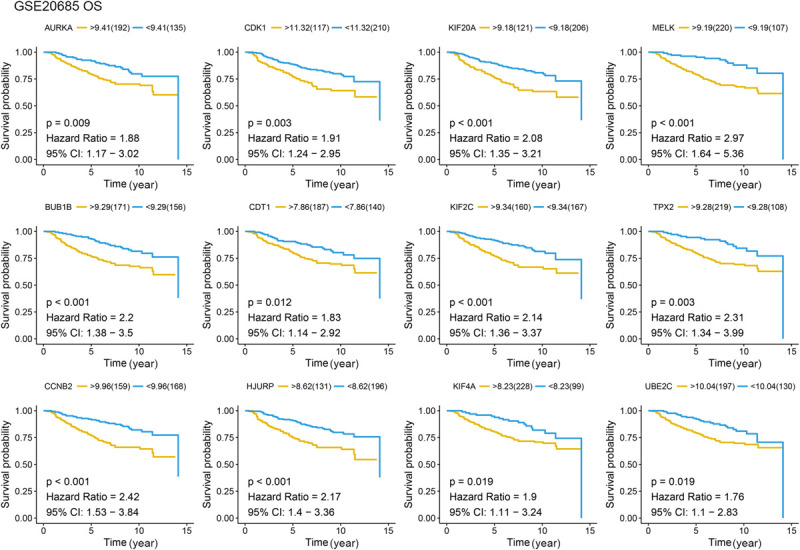
Overall survival (OS) analysis of the candidate hub genes. Overall survival analysis of the candidate hub genes based on GSE20685. The red line is the high gene expression group, and blue line is the low gene expression group. The unit of time is year.

**FIGURE 7:**
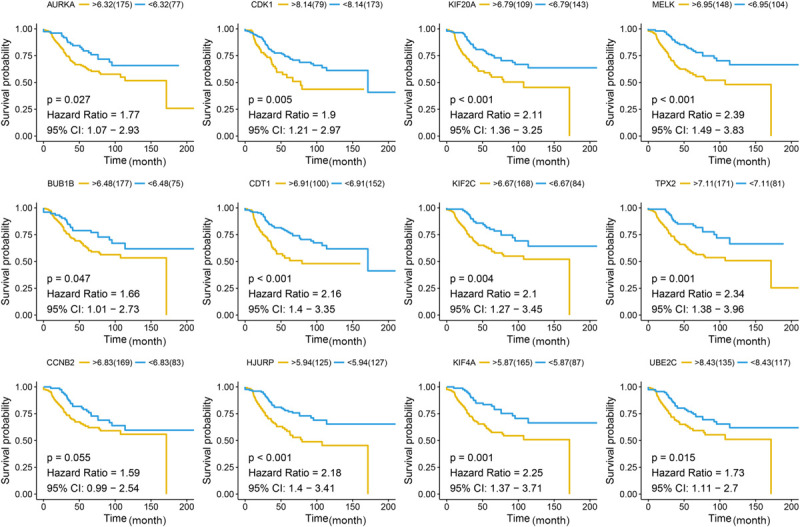
Disease-free survival (DFS) analysis of the candidate hub genes. Disease-free survival of the candidate hub genes based on GSE21653. The red line is the high gene expression group, and blue line is the low gene expression group. The unit of time is month.

Different methods were used to identify the hub gene from the hub module. Firstly, 504 genes in the stage-related module (blue module) are screened by module membership (MM) and gene significance (GS). As mentioned in section “Materials and Methods,” when the absolute value of MM is greater than 0.8 and the absolute value of GS is greater than 0.2, 36 hub genes were identified (Figure 3E). The PPI network showed that 62 genes with the top connectivity degree were identified as hub genes under the cutoff of confidence > 0.4 and connectivity degree of ≥ 4 (node/edge) (Figure 3D and Supplementary Figure S6). One-way ANOVA analyses were performed to validate candidate hub genes in the datasets GSE102484, GSE20685, and TCGA-BRCA, and 30 of 36 genes could be verified. As tumor prognosis was always affected by tumor progression, the candidate hub genes were validated by overall survival analysis (OS), recurrence-free survival analysis (RFS), and metastasis-free survival analysis (MFS), which showed that most of the hub genes had significant *P*-values in different test sets (Figures 3F, 6, 7 and Supplementary Figures S4, S5). We regarded the common genes with statistical significance in different methods to the candidate hub genes, and 12 genes were finally screened (AURKA, BUB1B, CCNB2, CDK1, CDT1, HJURP, KIF20A, KIF2C, KIF4A, MELK, TPX2, UBE2C) (Figures 4A–C). As we all know, MKi67 is a cell proliferation marker, and the correlation coefficient between the candidate hub gene and MKi67 was calculated by Pearson correlation. The results showed that the expression of 12 candidate hub genes was highly positively correlated with MKi67. In addition, BC samples with stronger KI67 IHC staining showed higher gene expression of hub genes (Figures 5A–C).

## Discussion

By 2019, about 268,600 invasive BC and 48,100 DCIS cases were diagnosed among American women, and 41,760 women will die of the disease. About 13% of women will be diagnosed with invasive BC (DeSantis et al., 2019). From 2009 to 2015, the 5 years survival rate for women diagnosed with BC was stage I: 98%, stage II: 92%, stage III: 75%, and stage IV: 27% (Marinac et al., 2016). Because TNM staging focuses on the anatomical information of the tumor, the disease progression and prognosis of BC patients cannot be fully evaluated. So, our study aimed to find biomarkers that could adequately predict BC progression and prognosis.

WGCNA has been widely used in the screening of biomarkers that predict disease progression (Chen L. et al., 2017). WGCNA is an algorithm for mining gene module information from expression profile analysis chips, and it has been widely used in gene expression profile data analysis (Langfelder and Horvath, 2008). In this method, a module is defined as a set of genes, where genes have similar expression trends in different physiological processes or different samples. After identifying gene modules with WGCNA, the correlation between gene modules and clinical characteristics such as tumor stage and grade is calculated. In this way, the gene modules most relevant to clinical features can be used to explore the main causes of tumor development. The characteristic of the scale-free network is that there are some nodes, the connectivity of these nodes is much higher than that of ordinary nodes, and the “few” nodes genes are defined as central genes (Niemira et al., 2019). Therefore, the study on the correlation between the module of interest and certain clinical features can be simplified to the correlation between the module of interest and the hub genes, so as to provide an important molecular basis for studying the mechanism of disease (Chen L. et al., 2019; Tian et al., 2019; Wang et al., 2019). By comparing different histological levels of BC, molecular targets have been identified to distinguish different stages of BC (Tian et al., 2020). We use systematic biology methods to identify specific biomarkers of BC based on a large number of samples. In our study, eight co-expression gene modules were determined by the dynamic tree cutting method. Correlation analysis shows that the blue module has the highest correlation with tumor staging, identifying the hub gene with the highest connectivity from the hub module. The functional annotations of clinical related modules suggest focusing on the cell proliferation related pathways, such as organelle fission, nuclear division, and chromosome segregation. WGCNA has been used to identify biomarkers related to BC progression. In comparation with recent studies, Tang et al. used WGCNA to screen several prognostic genes, including CCNB2, FBXO5, KIF4A, MCM10 and TPX2. These five genes are all related to cell division, which is consistent with our results. Among them, three genes are consistent with the results we found. However, the difference is that we have more BC samples to discover and validate, and our results are complementary to their findings. In addition, we have included more methods, including module preservation analysis and protein–protein interaction (PPI) to make our findings convincing.

In this study, 12 pathological hub genes (AURKA, BUB1B, CCNB2, CDK1, CDT1, HJURP, KIF20A, KIF2C, KIF4A, MELK, TPX2, and UBE2C) that are significantly related to the pathological stage were identified and verified, and significant differences can also be found in the expression value of each hub gene between different tumor stages and grades. Further verification also confirmed that the 12 hub genes were positively correlated with the progression of BC, and their expression was also related to the prognosis of BC patients. Aurora kinase A (AURKA), a member of the serine/threonine kinase family, plays an important role in mitotic cell division and genetic instability (Wu et al., 2018). It has been reported to stabilize FOXM1 by attenuating its ubiquitination in triple-negative breast cancer (TNBC), thus promoting proliferation of TNBC cells (Gartel, 2017). It has also been found to inhibit autophagy induction, suggesting that it may be the mechanism of drug-resistant BC cell apoptosis26. BUB1 mitotic checkpoint serine/threonine kinase B (BUB1B) encodes is a kinase involved in spindle testing. This protein plays a key role in the cell cycle (Lee et al., 2017). Its mRNA level has been found to be associated with intrachromosomal instability (Lee et al., 2017). Cyclin-dependent kinase 1 (CDK1) is a mitotic kinase, it mainly mediates tumor-related cell cycle defects, misregulated CDK1 may cause tumor cell proliferation and genome instability (Prevo et al., 2018). It has been reported that CDK1 could directly phosphorylate AMPK and promote the progress of BC (Galindo-Moreno et al., 2017). Other hub genes also play an important role in promoting cancer in BC.

There are still some limitations to our research. First, all data used in our study were based on publicly available datasets without validating in prospective clinical trials. Moreover, several important clinical factors, such as tumor size and lymph node metastasis, were not provided in these datasets. Finally, the mechanism between these gene signatures and BC recurrence still needs further experimental verification. In conclusion, through high-throughput screening and further screening by the WGCNA algorithm, we finally identified 12 hub genes that were significantly related to the progress and prognosis of BC after layers of validation.

## Data Availability Statement

The original contributions presented in the study are included in the article/Supplementary Material, further inquiries can be directed to the corresponding author/s.

## Ethics Statement

The studies involving human participants were reviewed and approved by the Ethics Committee of Jiangsu College of Nursing. The patients/participants provided their written informed consent to participate in this study.

## Author Contributions

GS and WY conceived and designed the study and contributed to the writing of the manuscript. GS, ZS, and YL performed the analysis procedures, analyzed the results, and contributed analysis tools. All authors reviewed the manuscript.

## Conflict of Interest

The authors declare that the research was conducted in the absence of any commercial or financial relationships that could be construed as a potential conflict of interest.

## Footnotes

1 http://www.ncbi.nlm.nih.gov/geo/

## Supplementary Material

Supplementary Figure 1 The flow chart of the study.

Supplementary Figure 2 Sample clustering and determining the soft threshold power in WGCNA. **(A)** Sample clustering before removing the outlier samples. Four outlier samples could be found with red marks and were removed from further analysis. **(B)** Scale-free fitting index analysis of different soft threshold power (β). **(C)** The average connectivity analysis of various soft threshold powers. **(D)** The distribution of connectivity when β is 4. **(E)** Check the scale-free topology when β is 4.

Supplementary Figure 3 Boxplots of hub genes at different grades in the GSE42568. The relative expression of hub genes at different tumor grades. One-way ANOVA was used to test statistical significance at different tumor grades. **p* < 0.05, ***p* < 0.01, ****p* < 0.001.

Supplementary Figure 4 Metastasis-free survival (MFS) analysis of the candidate hub genes. Metastasis-free survival analysis of the candidate hub genes based on GSE21653. The unit of time is year.

Supplementary Figure 5 Recurrence-free survival (RFS) analysis of the candidate hub genes. Recurrence-free survival of the candidate hub genes based on GSE42568. The unit of time is day.

Supplementary Figure 6 Protein–protein interaction (PPI) network of the genes in clinically significant modules.
