# Supplementary figures and images for: Identifying Biomarkers to Predict the Progression and Prognosis of Breast Cancer by Weighted Gene Co-expression Network Analysis
Source: Front Genet. 2020 Dec 17;11:597888. doi: 10.3389/fgene.2020.597888 (PMC7773894; doi:10.3389/fgene.2020.597888)

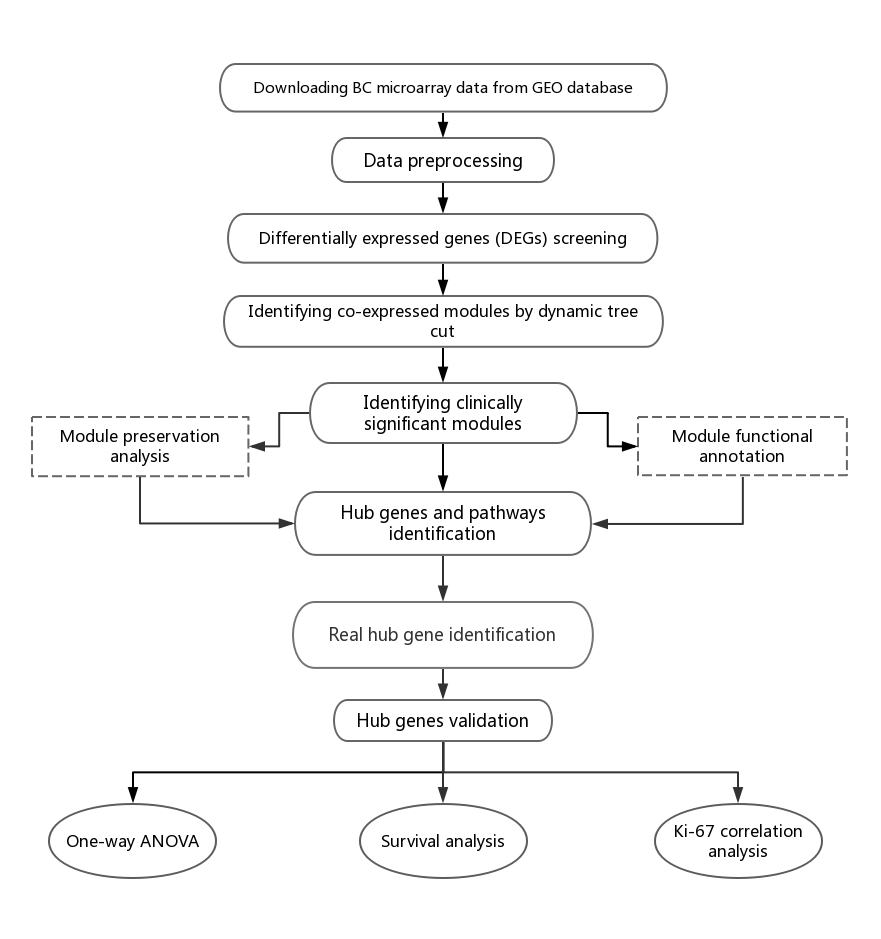

Supplement: Supplementary Figure 1 — The flow chart of the study. [file Image_1.TIF]

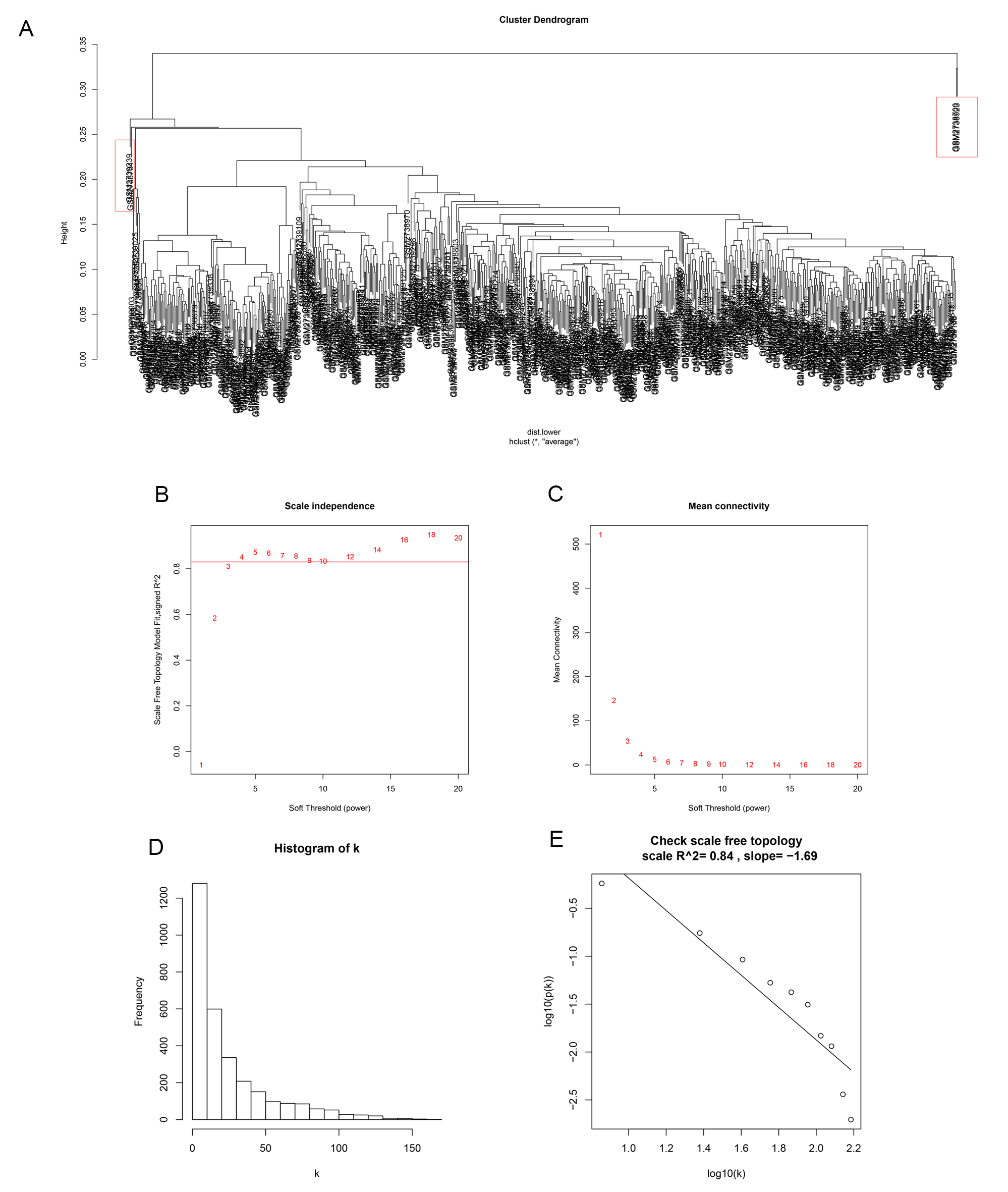

Supplement: Supplementary Figure 2 — Sample clustering and determining the soft threshold power in WGCNA. (A) Sample clustering before removing the outlier samples. Four outlier samples could be found with red marks and were removed from further analysis. (B) Scale-free fitting index analysis of different soft threshold power (β). (C) The average connectivity analysis of various soft threshold powers. (D) The distribution of connectivity when β is 4. (E) Check the scale-free topology when β is 4. [file Image_2.TIF]

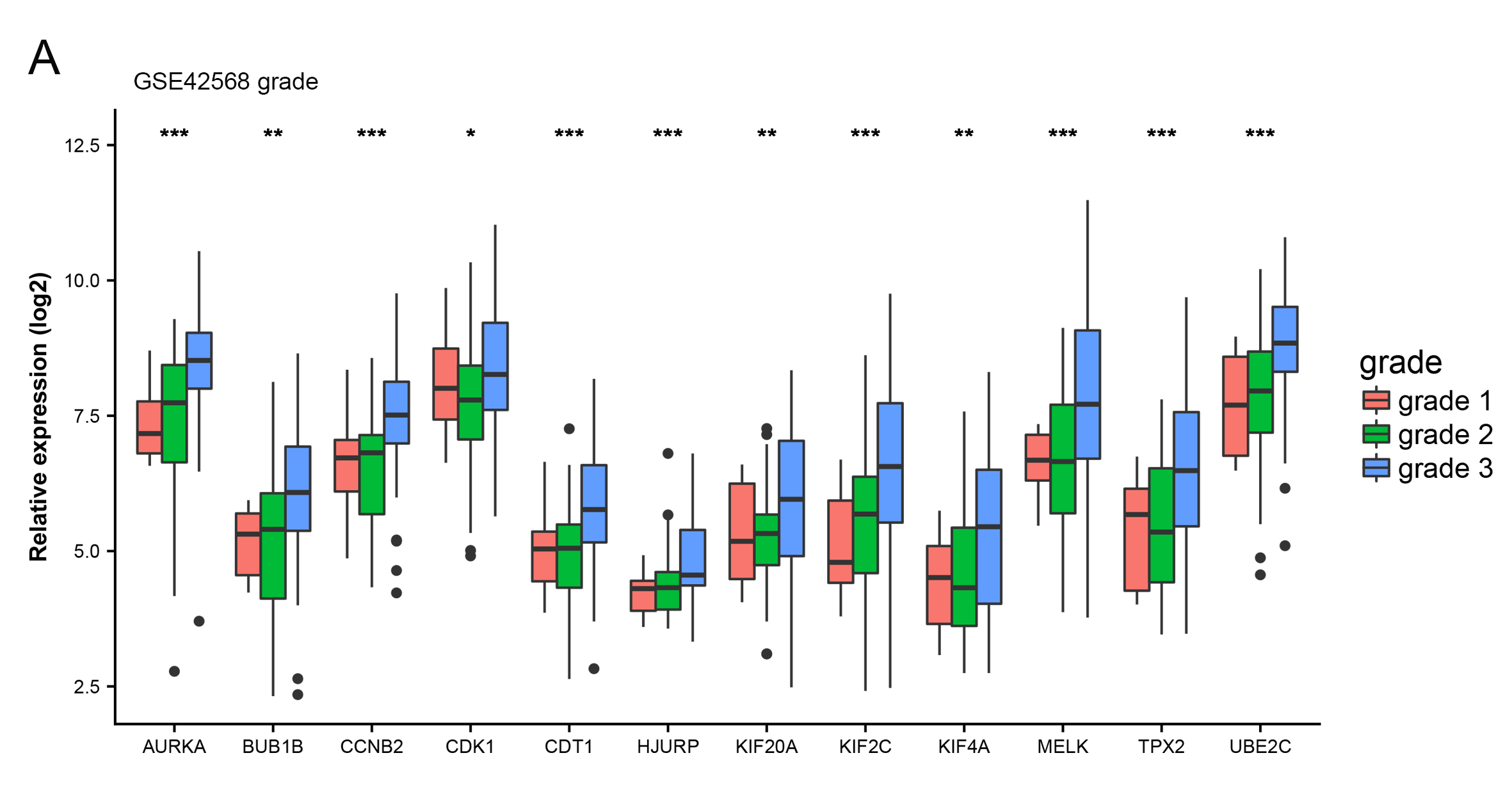

Supplement: Supplementary Figure 3 — Boxplots of hub genes at different grades in the GSE42568. The relative expression of hub genes at different tumor grades. One-way ANOVA was used to test statistical significance at different tumor grades. *p < 0.05, **p < 0.01, ***p < 0.001. [file Image_3.TIF]

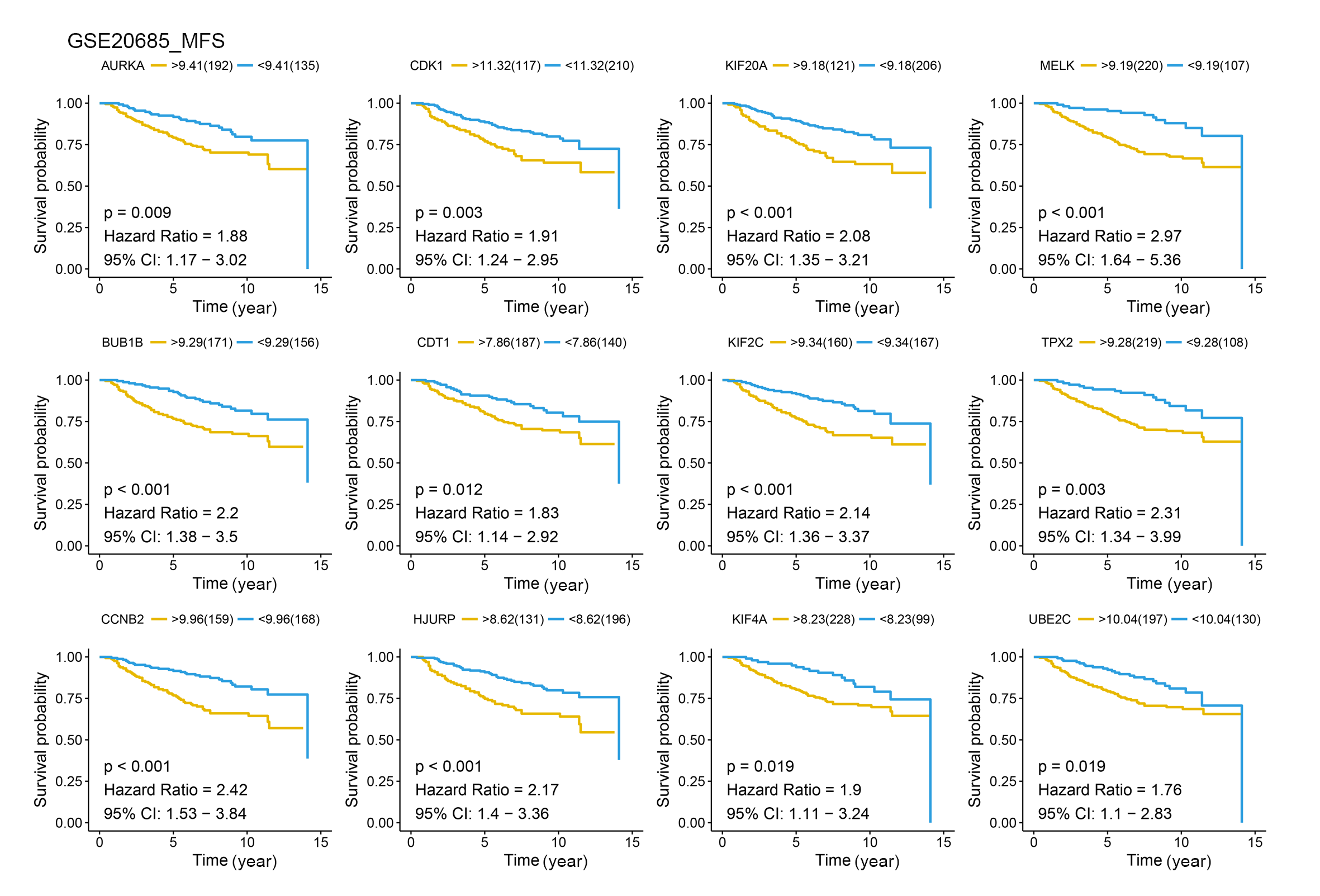

Supplement: Supplementary Figure 4 — Metastasis-free survival (MFS) analysis of the candidate hub genes. Metastasis-free survival analysis of the candidate hub genes based on GSE21653. The unit of time is year. [file Image_4.TIF]

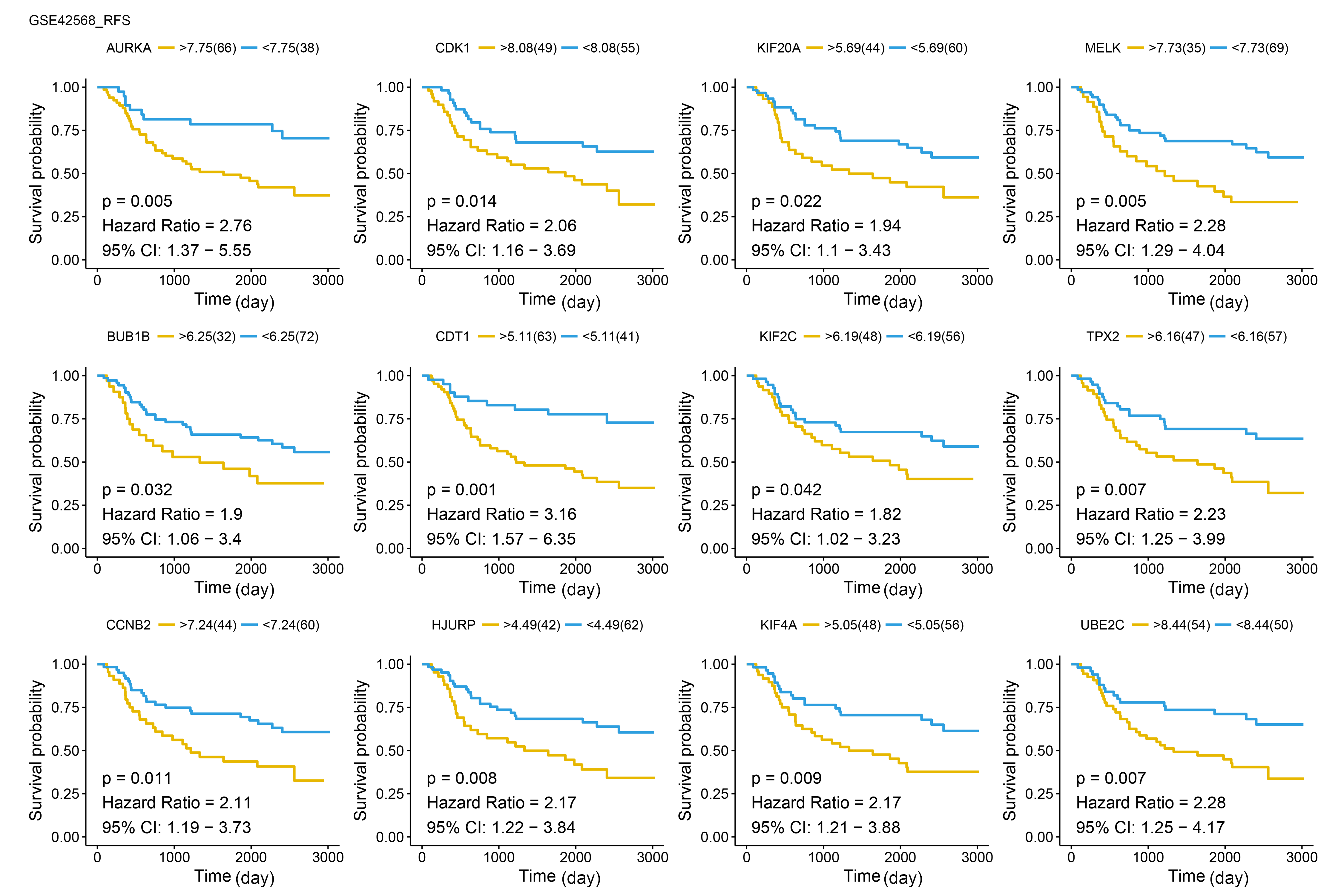

Supplement: Supplementary Figure 5 — Recurrence-free survival (RFS) analysis of the candidate hub genes. Recurrence-free survival of the candidate hub genes based on GSE42568. The unit of time is day. [file Image_5.TIF]

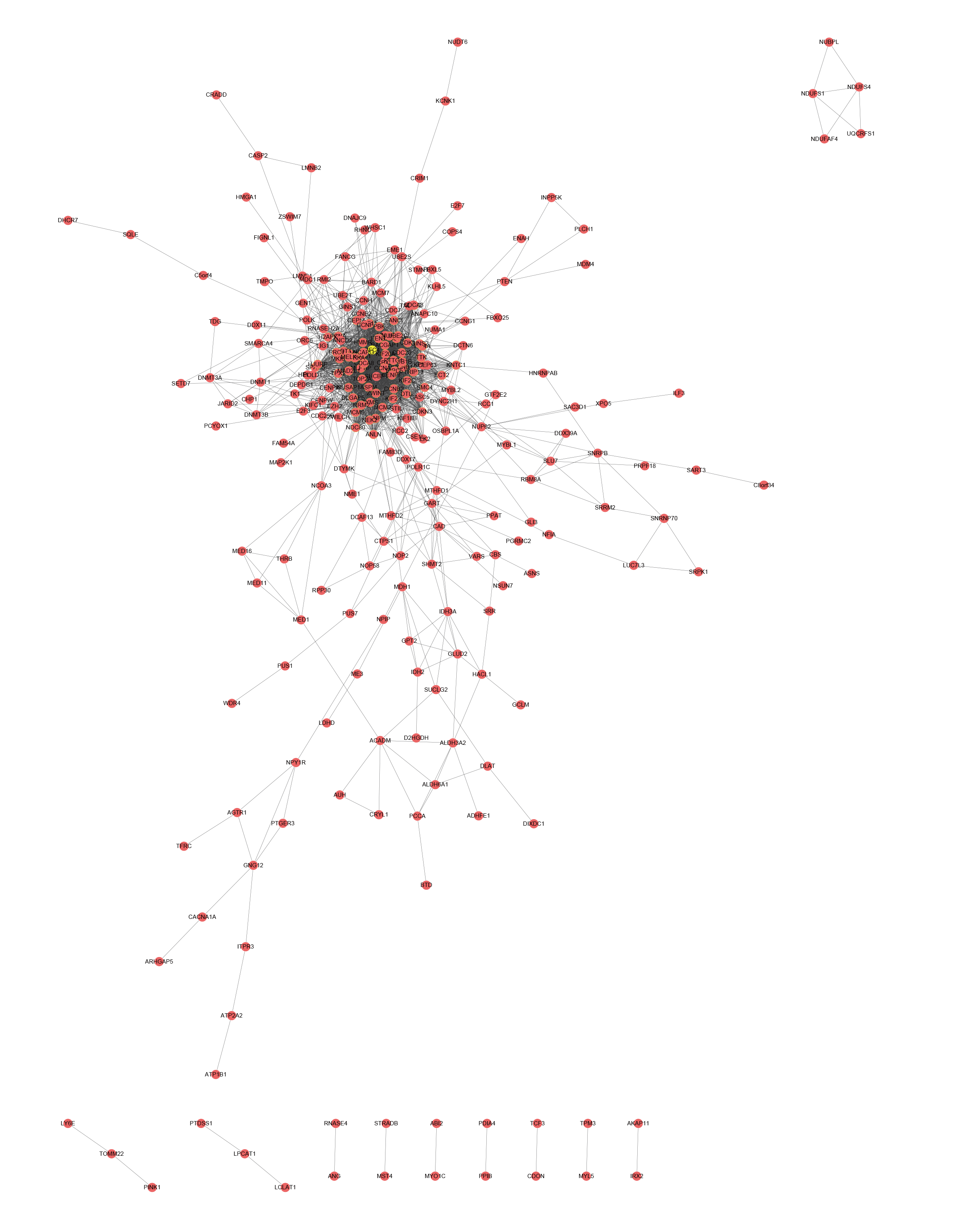

Supplement: Supplementary Figure 6 — Protein–protein interaction (PPI) network of the genes in clinically significant modules. [file Image_6.TIF]
